# The performance of the risk of ovarian malignancy algorithm

**DOI:** 10.1038/bjc.2011.224

**Published:** 2011-06-28

**Authors:** Z Langmár, M Németh, B Székely, G Borgulya

**Affiliations:** 1 2nd Department of Obstetrics and Gynecology, Semmelweis University, Budapest, Hungary; 2 Department of Gynecologic Oncology, St Stephen Hospital, Budapest, Hungary; 3 2nd Department of Pathology, Semmelweis University, Budapest, Hungary

**Sir,**

In a recent manuscript, van Gorp *et al* (2011) compared the diagnostic performance of serum tumour markers CA125 and HE4 and the ROMA clinical risk stratification tool in a prospective collection of serum samples from patients with ovarian mass. They reported statistically insignificant differences between these three diagnostic tests. However, the non-significance of the results may be due to lack of power. A weakness of the study is that the authors have not explained how they calculated the sample size and what the power of the study was. Van Gorp *et al* started enrolling patients in August 2005 and ended in March 2009. The first publication on the diagnostic performance of ROMA became available in 2008 (Moore *et al*, 2008), and there were no published papers on the diagnostic performance of either ROMA or HE4 in 2005. Therefore, it is unlikely that the authors had prior knowledge on the performance of these tests that could have been used for sample size and power calculations. Since enrolment and design of this prospective study preceded the publications of the tests that it intended to validate, this raises the question if the study originally was started as a generic pelvic mass diagnostic study and was re-interpreted as a validation trial after the first ROMA and HE4 publications came out. It is unclear from the publication if all enrolled patients were tested for HE4, CA125 and ROMA or only subsamples. Publication of the selection criteria, if any, is critically important, as it could lead to bias in the results. We think that the incomplete description of the study objectives and study population substantially weakens the conclusions. The non-significant differences in test performance were interpreted by van Gorp *et al* as equivalence in test performance. The authors stated that ‘HE4 and ROMA did not increase the detection of malignant disease compared to CA125 alone and neither HE4 nor ROMA increased the detection of malignant disease’. However, the AUC (95% CI) values for ROMA, CA125 and HE4 were 0.898 (0.863–0.926), 0.877 (0.840–0.908) and 0.857 (0.819–0.891), respectively, which shows a trend for better performance for ROMA. Approximate 95% confidence intervals (CIs) can be calculated from the data presented in the paper: AUC(ROMA)–AUC(CA125)=0.021 (−0.009 to 0.051), AUC(ROMA)–AUC(HE4)=0.041 (0.017 to 0.065), AUC(CA125)–AUC(HE4)=0.020 (−0.018 to 0.058). Therefore, about 5.1–6.5% AUC gain when using ROMA in place of CA125 or HE4 cannot be excluded with statistical certainty. Van Gorp *et al* concluded that measurement of HE4 serum levels does not contribute to the diagnosis of ovarian cancer. This conclusion could have relied on comparisons between AUC(CA125) and AUC(CA125 and HE4 combined) or AUC(CA125 and menopausal status combined) *vs* AUC(ROMA); however, such comparisons were not described in the paper. The AUC(ROMA)–AUC(CA125) comparison may be considered here, and according to the approximate 95% CI the gain may be as large as 5.1%, less the gain attributable to considering menopausal status. Van Gorp *et al* stated that even for the pre-menopausal patients, HE4 and ROMA did not perform better than CA125. Indeed, in this study among pre-menopausal patients, CA125 had a higher AUC than either HE4 or ROMA. These were, however, not significant, and the wide CIs did not even exclude the case that ROMA was 6.1% higher than CA125 in terms of AUC. Based on these considerations, a more appropriate conclusion would be the following: In this study ROMA had a higher AUC value discriminating between malignant and benign tumours than CA125 alone by 2.1% this difference was, however, not significant. A rough approximation of the 95% confidence interval of the AUC gain (−0.009 to 0.051) does not exclude a large 5.1% AUC gain when using ROMA in place of CA125 alone, but an AUC loss of 0.9% is also compatible with the data. In the pre-menopausal subgroup ROMA performed worse than CA125 alone, AUC difference −0.010 (rough CI: −0.081 to 0.061); the wide CI allows for both a substantial AUC gain and a substantial AUC loss.

A further important consideration is that the Van Gorp's study had a high cancer incidence (41.4%) rate in comparison with Moore's study (24.3%) (Moore *et al*, 2009) and a much higher proportion of postmenopausal women were included (∼74% in Van Gorp *vs* 53% in the Moore study). There were also differences in the distribution of histological types of tumours. Important differences that could account for the weaker performance of HE4 in the Van Gorp's study include higher proportion of mucinous tumours, LMPs and metastatic tumours of extra-ovarian origin, and lower number of serous tumours. There was also a small difference in Stage I/II *vs* Stage III/IV distribution, with more early-stage tumours in Van Gorp's study. Moore's study had a higher proportion of serous EOC (64.3 *vs* 52.2%) and more of the endometrioid type (12.4% *vs* only 4.3% in Van Gorp's study). These histological types tend to overexpress HE4 and contribute to favourable HE4 performance. Van Gorp's study had more mucinous tumours (13 *vs* 4.7% in Moore's study). This tumour type tends not to express HE4 and contributes to the lower performance of HE4. Van Gorp's study had a higher proportion of LMPs (23.7% *vs* 12.3% in Moore's study) and HE4 tends to lack the sensitivity to detect non-invasive LMP.

Van Gorp *et al* stated that combining HE4 and CA125 in ROMA improved HE4 but not CA125 performance. As CA125 is the current standard for comparison, this means neither HE4 nor the ROMA improved the diagnosis of ovarian cancer. This statement is also debatable, as it is not shown what percentage of patients had increased levels of CA125 in this study. If the majority of patients had increased CA125, in the first place no marker alone or marker combination could improve on it. The interpretation of the HE4 thresholds is also arguable: the optimal threshold depends on the characteristics of the population and the consequences of the true and false test outcomes; but in any case HE4 is not intended to be used for screening in a group comparable to the healthy controls, rather among patients with adnexal mass.

## References

[bib1] Moore RG, Brown AK, Miller MC, Skates S, Allard WJ, Verch T, Steinhoff M, Messerlian G, DiSilvestro P, Granai CO, Bast Jr RC (2008) The use of multiple novel tumor biomarkers for the detection of ovarian carcinoma in patients with a pelvic mass. Gynecol Oncol 108(2): 402–408 18061248 10.1016/j.ygyno.2007.10.017

[bib2] Moore RG, McMeekin DS, Brown AK, DiSilvestro P, Miller MC, Allard WJ, Gajewski W, Kurman R, Bast Jr RC, Skates SJ (2009) A novel multiple marker bioassay utilizing HE4 and CA125 for the prediction of ovarian cancer in patients with a pelvic mass. Gynecol Oncol 112(1): 40–46 18851871 10.1016/j.ygyno.2008.08.031 PMC3594094

[bib3] Van Gorp T, Cadron I, Despierre E, Daemen A, Leunen K, Amant F, Timmerman D, De Moor B, Vergote I (2011) HE4 and CA125 as a diagnostic test in ovarian cancer: prospective validation of the Risk of Ovarian Malignancy Algorithm. Br J Cancer 104(5): 863–870 21304524 10.1038/sj.bjc.6606092 PMC3048204

